# An Account of the Practice of One of the Physicians of the Westminster General Dispensary, and of the Western Dispensary, from the 20th of September, to the 20th of October, 1807

**Published:** 1807-11

**Authors:** Samuel Fothergill

**Affiliations:** Southampton-Street


*An Account of the Practice of one of the Physicians of the Westminster General Dispensary, and of the Western Dispensary, from the 20th of September, to the 20th of October,* 1807.

| ACUTE DISEASES. | |
|---|---|
| Synochus | 4 |
| Typhus | 1 |
| Scarlatina Anginosa | 2 |
| Scarlatina | 3 |
| Inflammatory Sore-throat | 2 |
| Ulcerated Sore-throat | 1 |
| Acute Rheumatism | 4 |
| Hepatitis | 1 |
| Small-pox | 5 |
| Chicken-pox | 1 |
| Measles | 3 |
| Enteritis | 1 |
| Catarrh | 2 |
| Gout | 2 |
| Acute Diseases of Infants | 12 |

| CHRONIC DISEASES. | |
|---|---|
| Pulmonary Consumption | 6 |
| Cough and Dyspnœa | 16 |
| Asthma | 2 |
| Hæmoptoe | 4 |
| Pleurodyne | 2 |
| Chronic Rheumatism | 7 |
| Lumbago | 3 |
| Cephalalgia and Vertigo | 5 |
| Asthenia | 10 |
| Paralysis | 3 |
| Dropsy | 4 |
| Gravel and Dysury | 3 |
| Stone | 1 |
| Gastrodynia | 6 |
| Enterodynia | 2 |
| Scirrhous Liver | 1 |
| Jaundice | 2 |
| Colica Pictonum | 1 |
| Constipation and Vomiting | 2 |
| Diarrhœa | 10 |
| Dyspepsia | 8 |
| Pyrosis | 2 |
| Worms | 3 |
| Scrophula | 2 |
| Scurvy | 1 |
| Hectica Senilis | 1 |
| Psora | 3 |
| Impetigo | 2 |
| Porrigo | 1 |
| Chlorosis | 2 |
| Amenorrhœa | 3 |
| Menorrhagia | 2 |
| Leucorrhœa | 2 |
| Prolapsus Uteri | 2 |

Since the last Report, the bowel complaints, which were more than usually prevalent, have declined. Several children have been affected with Scarlatina, Measles, and Small-pox. The cases of the latter occurred to the children of poor people; they received the infection naturally, and two out of five, whom I attended, died. The usual fatality of this severe and loathsome distemper amongst the poor in this great town, is not to be wondered at. When we consider their general neglect of all precautionary means; their aversion from inoculation; and the wretched state in which they are generally exposed to the infection; their vitality being reduced to the lowest degree by the influence of filth, noxious air, and unwholesome diet; it is rather matter of surprise when they get through, than when they sink under, this formidable disorder. If the benefits of vaccination are generally diffused over the inhabited habited globe, they are surely most vitally felt in a close and crouded city: among the higher ranks of society, indeed, where every and immediate attention is paid, the ravages of small-pox are scarcely known; but when these are communicated to the poor inmates of the garret and the cellar, the fatality is immense; and humanity rejoices that, at length, this wide-wasting pestilence may be checked, and no longer be destructive to society. Vaccine inoculation, however, is yet far from being popular; and the coercive means which some gentlemen have thought proper to advise, and some to adopt, are ill calculated to make it so; let its advantages be manifested, and the question be fairly and candidly stated, and the public mind will gradually become satisfied, and acquiesce in the utility of a practice, which, if found to be certain, and permanently tenable, must make its way.

I am happy to record the successful termination of a case of Hydrocephalus Internus; a disease so frequently fatal to children, that when we hear of their recovering from it, we are inclined to doubt their having laboured under that complaint. The child was at the breast, about seven months old, and had been ill several weeks before I saw it, the 17th of last July, when it was considered as a lost case. The head was large, the sutures being considerably separated: pressure upon the superior fontanelle, occasioned immediate distress: the chin rested upon the chest, the child being wholly incapable of raising its head. The limbs were emaciated, and the inferior extremities drawn up towards the abdomen; the eyebrows were contracted; the pupils dilated; and, for some time, the little sufferer was quite insensible to light. He frequently screamed, as if in great pain; was always cross and fretful; and had disturbed sleep, suddenly starting and crying. The bowels were pretty regular, and he generally took the breast well. From his great restlessness and struggling, whenever I attempted to feel his pulse, I never was able to ascertain its frequency. The treatment was simple. A blister was applied over the whole head, and repeated four times, with good effect. One grain of calomel, with the sixth of a grain of opium, was given every four hours. On the 1st of August, the symptoms having rather increased than diminished, in addition to the above, I ordered a draught, with four drops of tincture of digitalis, as many of tincture of cinnamon, and one drop of laudanum, to be given also every four hours. In the space of a week, we began to entertain hopes of his recovery; and, by the 25th of August, he was so much better, that I

directed directed the medicines to be taken only three times a day. On the 12th of September he took them twice a day, gaining flesh fast; and, by the beginning of this month, he was quite strong, and had a healthy appearance, though not entirely free from complaint. On the 14th, the medicines were discontinued, and he now remains entirely well. I omitted to state, that the bowels were occasionally purged with scammony and calomel, and that no worms were evacuated. The head still appears large, in proportion to the body; but the child expresses no uneasiness, when pressure is made upon the fontanelle. Though the calomel was continued nearly three months, the mouth was not in the least affected, and the bowels very seldom. The stomach generally retained the digitalis draught, and I never perceived any ill effect follow its use.

SAMUEL FOTHERGILL.

*Southampton-Street, Oct. 26,* 1807.

